# Proteomic profiling of human menisci from mild joint degeneration and end-stage osteoarthritis versus healthy controls

**DOI:** 10.1016/j.ocarto.2023.100417

**Published:** 2023-11-18

**Authors:** Rocío Paz-González, Aleksandra Turkiewicz, Neserin Ali, Cristina Ruiz-Romero, Francisco J. Blanco, Martin Englund, Patrik Önnerfjord

**Affiliations:** a Grupo de Investigación de Reumatología (GIR), Unidad de Proteómica. INIBIC-Hospital Universitario A Coruña, SERGAS, 15006, A Coruña, Spain; b Grupo de Reumatología y Salud, Departamento de Fisioterapia y Medicina. Centro de investigaciones Avanzadas (CICA), Universidad de A Coruña, A Coruña, Spain; c Centro de Investigación Biomédica en Red de Bioingeniería, Biomateriales y Nanomedicina (CIBER-BBN), Spain; d Clinical Epidemiology Unit, Orthopedics, Clinical Sciences Lund, Lund University, Lund, Sweden; e Rheumatology and Molecular Skeletal Biology, Clinical Sciences Lund, Lund University, Lund, Sweden

**Keywords:** Meniscus, Osteoarthritis, Degeneration, Proteomics

## Abstract

**Objective:**

To gain new insight into the molecular changes of the meniscus by comparing the proteome profiles of healthy controls with mild degeneration and end-stage osteoarthritis (OA).

**Method:**

We obtained tissue plugs from lateral and medial menisci of 37 individuals (central part of the posterior horn) classified as healthy (n ​= ​12), mild signs of joint damage (n ​= ​13) and end-stage OA (n ​= ​12). The protein profile was analysed by nano-liquid chromatography-mass spectrometry using data-independent acquisition and quantified by Spectronaut. Linear-mixed effects modelling was applied to extract the between-group comparisons.

**Results:**

A similar protein profile was observed for the mild group as compared to healthy controls while the most different group was end-stage OA mainly for the medial compartment. When a pattern of gradual change in protein levels from healthy to end-stage OA was required, a 42-proteins panel was identified, suggesting a potential role in OA development. The levels of QSOX1 were lower and G6PD higher in the mild group following the proposed protein abundance pattern. Qualitative protein changes suggest lower levels of CYTL1 as a potential biomarker of early joint degradation.

**Conclusion:**

For future targeted proteomic approaches, we propose a candidate panel of 42 proteins based on gradually altered meniscal posterior horn protein abundance patterns associated with joint degradation.

## 1 Introduction

Traditionally, articular cartilage degeneration has been considered the hallmark of OA. However, recent studies describe knee OA as a whole-joint disorder, highlighting the important contribution of the meniscus in joint degradation [1]. The major components of the meniscal matrix are collagens type I and II, proteoglycans and adhesion glycoproteins, although deeper knowledge about the distribution of less abundant proteins in the meniscus is still limited. The relationship between meniscus and OA is complex and not fully elucidated. Nonetheless, the loss of meniscal function, by injury or degenerative processes, is one of the strongest risk factors associated with knee OA [2]. The slow degradation of meniscal tissue has been suggested to occur in the early stages of OA, but little is known about the underlying molecular processes involved [3].

There is a need to clarify the role of the meniscus in knee OA which may be aided by the identification of biomarkers that reflect the molecular events involved in tissue degradation [4]. This is particularly important in the early stages, a crucial phase when it might still be possible to interfere with disease progression before tearing and critical loss of meniscal function occurs. One way to achieve this goal is exploratory proteomics by mass spectrometry (MS), which has the potential to analyse the proteome of complex samples [5]. Our hypothesis was that there are differences in the protein profile of subjects with mild signs of joint damage compared to healthy individuals. The identification of quantitative changes in protein levels following a gradual trend of abundance depending on the degree of joint damage may elucidate the role of the menisci in OA onset.

Thus, our aim was to gain new knowledge about the molecular processes within the human meniscus by comparing proteomic profiles of both the lateral and medial meniscus from healthy controls, subjects with mild signs of joint damage, and patients with end-stage knee OA, using a non-targeted MS approach operating in the cutting-edge data-independent acquisition (DIA) mode.

## 2 Methods

### 2.1 Patients and meniscal tissue selection

This work was carried out using human meniscal tissue samples provided by the biobank at Skåne University Hospital, Lund, Sweden following approval by the regional ethics committee in Lund (2015/39 and 2016/865).

Full depth tissue plugs were obtained from the central part of the posterior horns of both lateral and medial menisci from the right knee of 37 individuals classified as 1) controls (deceased donors without known clinical knee OA or rheumatoid arthritis and without macroscopic signs of cartilage and/or meniscus degeneration), 2) mild joint damage (deceased donors without known clinical knee OA but with macroscopic signs of cartilage and/or meniscus degeneration in the medial compartment), or 3) end-stage medial knee OA. For the end-stage knee OA group the menisci were retrieved during total knee replacement (TKR) surgery at Trelleborg Hospital. The knee joint cartilage was classified by the surgeon according to the Outerbridge classification system. To be eligible as an end-stage knee OA patient, the highest Outerbridge grade of IV was required for the medial compartment, whereas the grade was required to be lower in the lateral compartment. The meniscal and cartilage macroscopic classification for each patient is detailed in Supplementary table 1.

For the research purpose six groups were established: medial and lateral menisci from control donors, medial and lateral menisci from donors with mild signs of joint damage and medial and lateral menisci from end-stage knee OA patients leading a total of 74 menisci samples. The groups for comparison will hereafter be referred to as control^med^ (n ​= ​12), control^lat^ (n ​= ​12), mild^med^ (n ​= ​13), mild^lat^ (n ​= ​13), end-stage OA^med^ (n ​= ​12) and end-stage OA^lat^ (n ​= ​12), respectively. The median age and the body mass index (BMI) of the study participants are balanced between the different study groups (Table 1).

**Table 1:** Characteristics of the study participants.

	Healthy Controls (n ​= ​12)	Mild joint damage (n ​= ​13)	End-stage OA (n ​= ​12)
Age, mean (SD)	68.8 (10.9)	70.5 (8.8)	72.4 (5.5)
BMI, mean (SD)	26.4 (5.9)	26.9 (5.8)	28.2 (4.5)
% women	42	38	33

BMI: body mass index; SD: standard deviation; OA: osteoarthritis.

### 2.2 Preparation of meniscal tissue for MS analysis

The complete menisci were thawed in phosphate-buffered saline (PBS). To prepare the tissue samples, a plug of 3 ​mm diameter was punched vertically through the central part of the posterior meniscus horn and stored at −80 ​°C until further analysis.

For protein extraction, the tissue plugs were powdered in liquid nitrogen using a pestle and mortar technique. After protein extraction, samples were reduced and alkylated prior to the tryptic digestion. Before MS analysis, samples were filtered and desalted. The protocol followed for protein extraction and digestion is detailed in the Supplementary Methods.

### 2.3 Mass spectrometry analysis

Dried samples were reconstituted in 50 ​μL of 0,1 ​% FA and 1 ​μL of each was injected using a blocked randomization sequence to an Easy nano-LC 1000 HPLC system (Thermo Fisher Scientific) equipped with an Acclaim PepMap® 100 nanoViper pre-column (Thermo Scientific, C18, 3 ​μm particles, 75 ​μm i.d. 2 ​cm long) and a PepMap® RSLC C18 analytical column (Thermo Scientific, C18, 2 ​μm particles, 75 ​μm i.d. 25 ​cm long) and analysed using a Q-Exactive™ ​HF-X quadrupole Orbitrap benchtop mass spectrometer (Thermo Fisher Scientific) operating in DIA mode. The settings of the LC-MS/MS method are listed in the Supplementary Methods.

The system was controlled by the Xcalibur™ Software (Thermo Fisher Scientific). Blank runs were injected between every sample to avoid cross-contamination. To verify the instrument performance a quality control sample (HeLa Protein Digest Standard) was injected every 10th injection. One sample from each of the six groups was randomly selected to be run twice for reliability assessment.

### 2.4 Data analysis

The DIA raw data generated from the meniscal samples were converted to HTRMS format using the tool HTRMS Converter (Biognosys AG, Switzerland).

For the quantitative data analysis, a spectral library was created in Spectronaut X™ Pulsar (version 14, Biognosys AG) based on the DIA data. A total of 94 DIA runs (74 menisci samples, 6 duplicates and 14 samples run twice in varying dilutions) were loaded in Spectronaut™ Pulsar and the protein search was performed using the created spectral library and the human protein data base downloaded from Uniprot 20210115_ UP5640 containing 20379 proteins). For protein identification an FDR< 0.01 was stablished in Spectronaut both at protein and peptide level.

Default settings (BGS factory settings) were used for the search with additional modifications: cysteine carbamidomethylation as a fixed modification, and N-terminal acetylation and methionine oxidation as variable modifications. The precursor quantification was performed at MS2 level, and the peak area of the top three proteotypic peptides for each protein was used for quantification. Cross-run normalization was performed using global normalization on median peptide intensity. The mass spectrometry proteomics data have been deposited to the ProteomeXchange Consortium via the PRIDE [6] partner repository with the dataset identifier PXD042122.

### 2.5 Statistical analysis

All run samples were included in the statistical analysis models (including the technical replicates and samples run in varying dilutions) after normalization. Peptide quantification data was transformed using log_2_ prior to the analysis and peptide level data was employed including only the peptides of good quality as selected by “top3” methods in Spectronaut. Linear mixed effects models were used with group, compartment, and their interaction as well as peptide as fixed effects and random intercepts for compartment and person (compartments nested within persons) to account for correlation of data coming from the same individual (and same meniscus). Each protein was analysed in a separate model and a protein was deemed suitable for analysis if at least 7 valid values were available in each group and compartment. The between-group comparisons in protein quant were extracted from the models, separate for medial and lateral compartment. After transforming back, these estimates are ratios of geometric means that in the results section are referred to as fold changes. We calculated the FDR q-values for each protein, separate for each compartment. We used the method of Yekutieli using package qqvalue in Stata 18.

To minimize loss of information due to missing data, proteins with a clear pattern of missing values were assessed. The proteins selected for manual check fulfil the criteria of being detected in 10 or more subjects from at least one group and detected in 2 or less samples from another group. An overview of missingness patterns was reported for each of these proteins.

### 2.6 Pathway enrichment analysis

The pathway enrichment analysis was performed in STRING (v 11.5) based on Gene Ontology (GO), KEGG, WikiPathways and Reactome databases to build a protein-protein interaction network and explore the biological process associated with joint damage in OA [7]. The enrichment analysis was performed with the total of proteins that follow a “protein abundance pattern” based on a gradual change in protein levels depending on the degree of joint damage from healthy to end-stage OA in the medial compartment crossing mild joint damage groups. We also considering the direction that changes in protein levels follow into the pattern (increased or decreased in end-stage OA with healthy as reference). First, the whole network was clustered applying the k-means clustering option (k ​= ​2). Then, we explore the most strongly represented pathways in each cluster.

## 3 Results

We identified a total of 2473 proteins in the meniscal tissue. Given that over 1000 proteins were statistically analysed, we report results for all of them in appendix (Supplementary table 2). Further, we applied filtration criteria to identify potentially interesting proteins. First, we identified 106 proteins with no difference between any of the groups defined by a confidence interval (CI) enclosed between −1 and 1 (log_2_) for all comparisons (Supplementary table 3). In addition, we identified 213 proteins with a CI too wide to provide any useful information (CI width larger than 2.5 (log_2_) in at least one comparison). These proteins offer inconclusive results due to a high variability (Supplementary table 4).

**Fig. 1:**
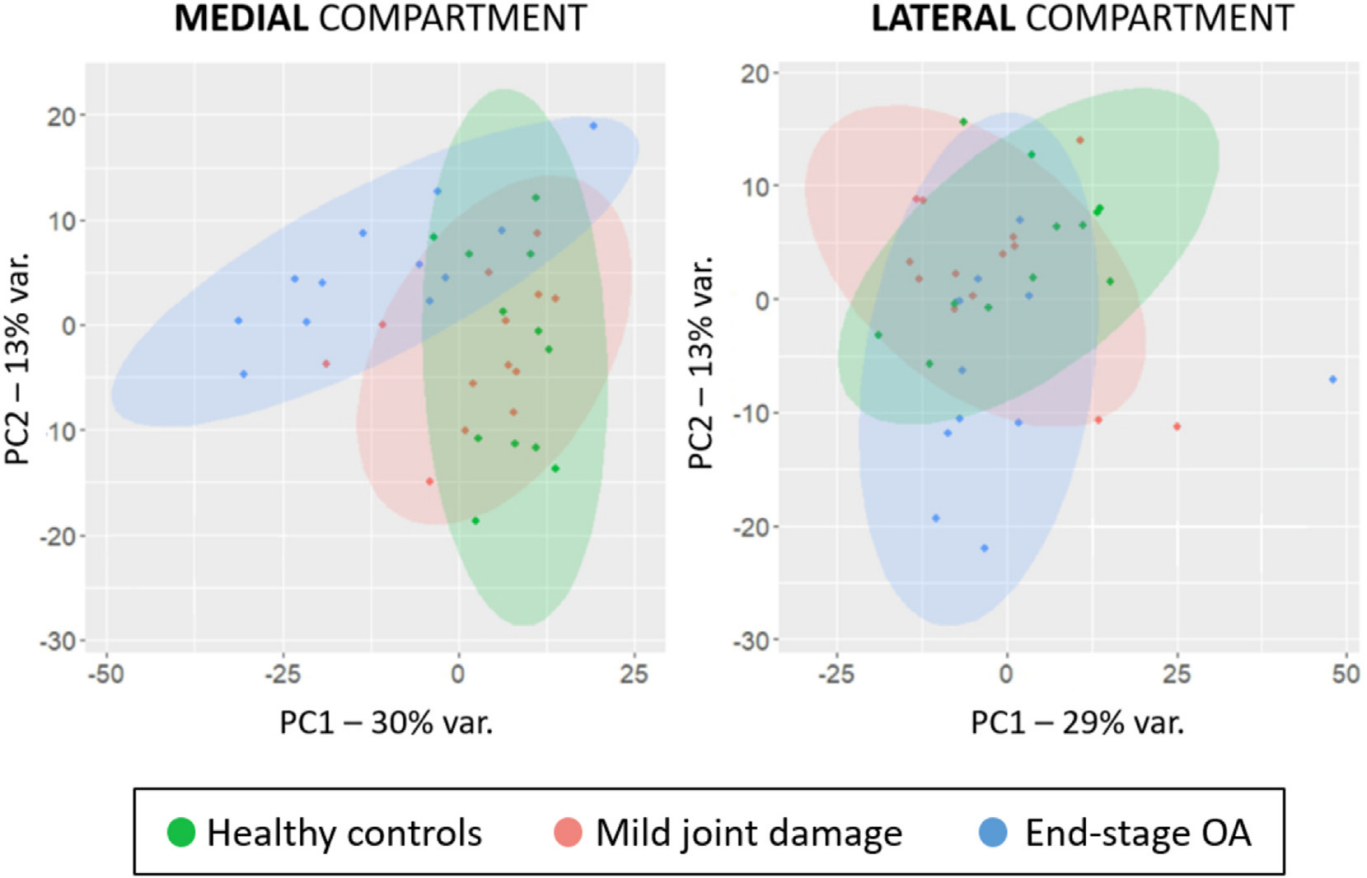
PCA plots for the different sample groups in medial (left) and lateral (right) meniscal compartments.

This filtration leads to a total of 777 proteins potentially regulated in either end-stage OA or mild joint damage groups in comparison to controls (Supplementary table 5). The metrics for each comparison are reported as a fold change on log_2_ with 95 ​% CI and *p-value*. The differences between the groups for each compartment are visualized by PCA-plots (Fig. 1). We discerned the end-stage OA group, mostly for the medial compartment, as the most different group as compared with healthy controls. In terms of changes in protein levels (fold change (log_2_) ​≥ ​1 and a p-value< 0.05) we found the largest differences for the end-stage OA^med^ group regard to the healthy controls followed by end-stage OA^lat^ whit only 25 proteins in common. To assess the strength of our results, we compared our findings with previous results from an independent study (Supplementary table 6) [8].

In the PCA plot, we also observed that the mild joint damage group mostly overlaps with the healthy control for both compartments. The group with less changes in protein levels (fold change (log_2_) ​≥ ​1 and a p-value< 0.05) was mild^lat^ where only glypican-6 (GPC6) and complement C1q tumour necrosis factor-related protein 8 (C1QTNF8) were increased. In the mild^med^ group the sulfhydryl oxidase 1 (QSOX1) levels decreased whereas the levels of three proteins increased: T-complex protein 1 subunit zeta (CCT6A), eukaryotic translation initiation factor 4B (EIF4B), and spermidine synthase (SPEE). The metrics for these proteins are summarized in Table 2.

**Table 2:** Proteins altered in the mild joint damage group as compared to healthy controls. Proteins which levels are regulated (fold change of ≥2 or ≤0.5 and a p-value <0.05) comparing the human menisci with mild signs of joint damage and healthy controls.

Mild group vs control	Gene Name	Protein ID	Protein name	Fold change (CI-95 ​%)	p-value
Medial compartment	QSOX1	O00391	Sulfhydryl oxidase 1	0.48 (0.25, 0.924)	0.029
	CCT6A	P40227	T-complex protein 1 subunit zeta	2.121 (1.132, 3.978)	0.020
	EIF4B	P23588	Eukaryotic translation initiation factor 4B	2.407 (1.302, 4.451)	0.006
	SRM	P19623	Spermidine synthase	2.412(1.236, 4.701)	0.011
Lateral compartment	GPC6	Q9Y625	Glypican-6	2.034 (1.035, 4.000)	0.040
	C1QTNF8	P60827	Complement C1q tumor necrosis factor-related protein 8	2.076 (1.006, 4.290)	0.048

CI: confidence interval.

### 3.1 Protein abundance pattern

**Fig. 2:**
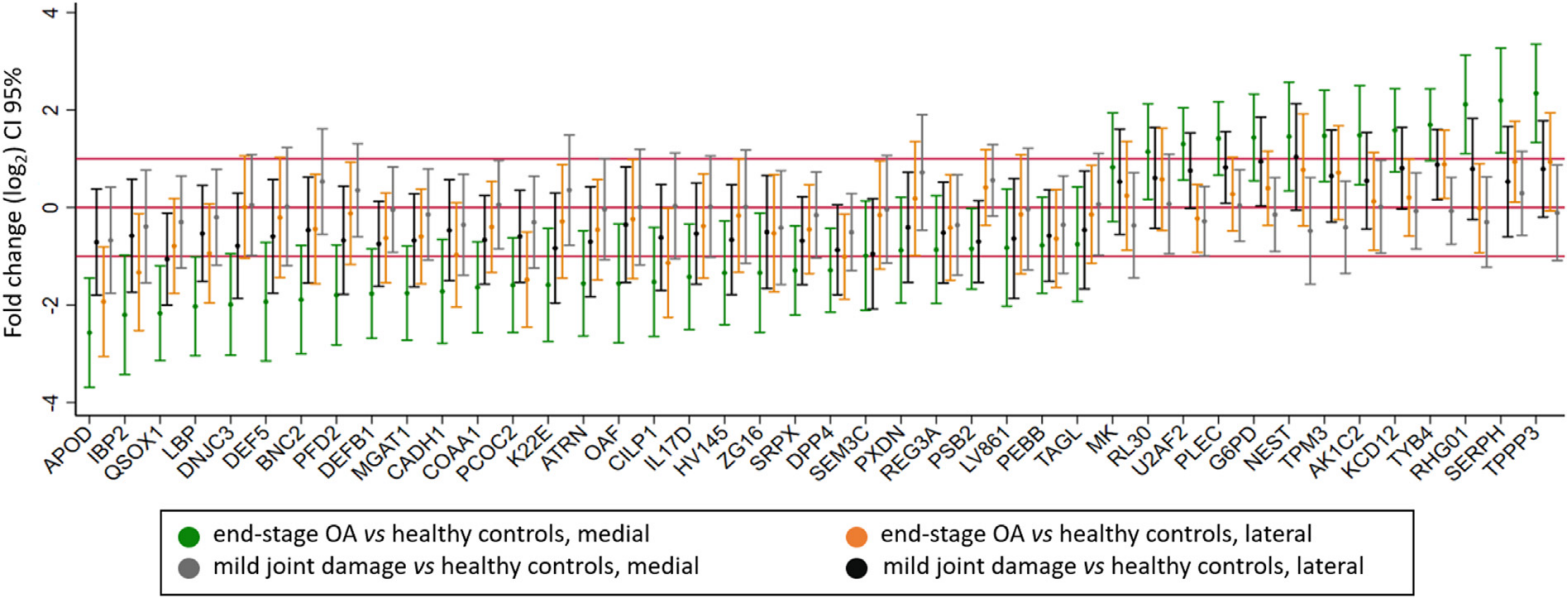
Proteins following the protein abundance pattern of gradual change in protein levels from the end-stage OA^med^ group representing the most advanced disease status to the mild^lat^ group as the least diseased compared to healthy controls. Only proteins with relevant changes in mild^med^ (value of 1.5 in the CI) as compared to healthy are illustrated (N ​= ​42).

A further selection among the 777 proteins potentially regulated was carried out to highlight the proteins that could be involved in the development of knee OA at early disease stages. To this end, we considered a protein to exhibit a “protein abundance pattern” when the difference in protein levels was largest (in absolute terms) between the end-stage OA^med^ vs healthy^med^, while smallest was between mild^lat^ vs healthy^lat^, with the other group comparisons in-between. In this first approach, we found 247 proteins following the protein abundance pattern above described. Then, to highlight the changes in the mild group, we restricted the protein abundance pattern to report only those proteins where CIs included values of 1.5 (log_2_) or larger specifically in the mild^med^ group. According to these criteria, 13 proteins were found to be increased and 29 decreased (Fig. 2). Among them, only the levels of thymosin beta-4 (TMSB4X), plectin (PLEC), G6PD, and QSOX1 clearly change in the mild^med^ group.

**Fig. 3:**
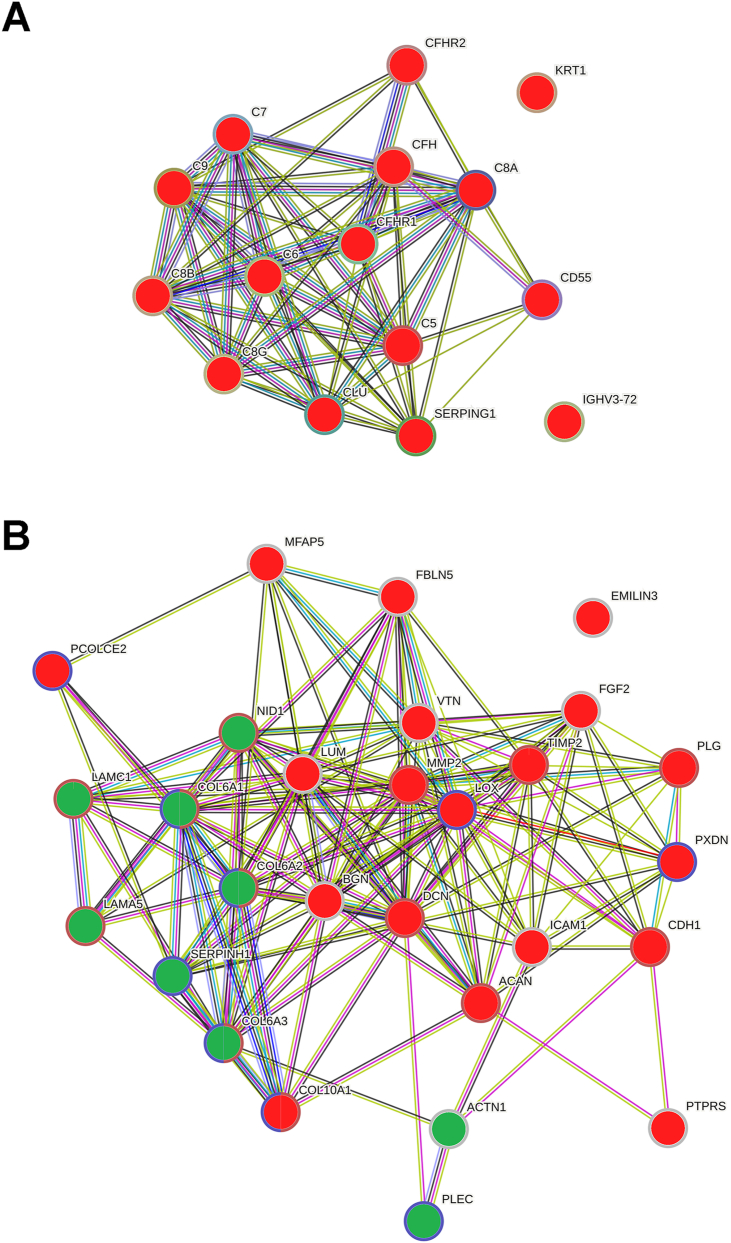
**Pathway enrichment analyses performed in STRING.** Proteins following the protein abundance pattern were clustered (k ​= ​2) to explore their biological function. A) Proteins related to the complement cascade, the most represented pathway of cluster 1. B) Proteins related to the ECM reorganization, the most represented pathway of cluster 2. Red: Proteins decreased following the protein abundance pattern. Green: Proteins increased following the protein abundance pattern.

We identified in STRING two independent cluster in the set of proteins following the protein abundance pattern (n ​= ​247) (Supplementary table 7). In the first cluster, the pathways enriched were related to the immune system, mainly to the complement system. All the proteins associated with the complement system were found decreased following the protein abundance pattern (Fig. 3A). The second cluster was comprised of proteins involved in the ECM organization (Fig. 3B). In addition, we focused on the pattern exhibited by the groups of proteins most relevant in the meniscus to evaluate their link with the biological processes identified in STRING in relation to knee OA physiopathology. The profile of the identified ECM family proteins, small leucine-rich proteoglycans (SRLP), proteases and proteases inhibitors were retrieved. In this section, we found proteins levels of prolargin (PRELP) and cartilage intermediate layer protein 1 (CILP) decreased and tenascin (TNC) increased following the trend defined by the protein abundance pattern. We also report levels of cartilage acidic protein 1 (CRTAC1) (fold-change(log2) [CI 95 ​%] ​= ​−0.975 [−1.606, −0.345]), aggrecan core protein (ACAN) (fold-change(log2) [CI 95 ​%] ​= ​−1.309 [−2.510, −0.107]), biglycan (BGN) (fold-change(log2) [CI 95 ​%] ​= ​−0.967 [−1.778, −0.155]) and decorin (DCN) (fold-change(log2) [CI 95 ​%] ​= ​−1.280 [−2.101, −0.459]) decreased and collagen IV (CO6A1, CO6A2 and, CO6A3) (fold-change(log2) ​= ​0.798, 1.325 and, 1.155 respectively, p-value <0.05) increased in the meniscus of end-stage OA patients following the protein abundance pattern but with minor differences in the mild joint damage group as compared to healthy.

### 3.2 Qualitative differences

Most of the proteins exhibited a higher number of missing values in the control group and the percentage decreased with the increment in tissue degeneration, being the end-stage OA group where a minor number of missing values were found (Supplementary Table 8). The clearest examples of qualitative changes are the proteins S100-A8 (S100A8) and cytokine-like protein 1 (CYTL1). S100A8 was identified in almost all end-stage OA samples, and completely missing in the control group. In contrast, CYTL1 was detected in most of the control and mild samples but this dramatically decreased in the end-stage OA group.

## 4 Discussion

We explored the proteome of human menisci obtained from subjects with different degrees of cartilage and/or meniscus degeneration by MS using a DIA approach [9]. We found a pronounced alteration of the meniscal proteome between the individuals with severe joint degeneration compared with the healthy controls, whereas only minor differences were found in those with mild signs of joint damage (see also Fig. 1). However, we proposed a panel of proteins which levels gradually change with the joint damage severity from healthy to end-stage OA crossing mild joint damage group to explore the role of meniscus in the disease (see also Fig. 2).

Accordingly to previous results [8], the largest differences were found comparing the controls with the end-stage OA group in the medial compartment. One of the largest differentially expressed proteins in end-stage OA for both compartments was apolipoprotein D (APOD) that was up to 2.6-fold decreased in OA as compared to healthy controls. APOD is a glycoprotein mainly related to lipid metabolism, but antioxidant and anti-inflammatory properties have also been described [10]. Previous studies correlated lower levels of APOD in cartilage, synovium, and serum with the severity of knee OA [11]. Although the correlation of APOD with OA is consistent, the underlying mechanism of APOD and apolipoproteins overall with knee OA progression is still unclear.

In the end-stage OA group, the levels of 25 proteins changed in common for both compartments and all of them decreased as compared to the healthy controls. Most of these proteins have been described in the OA process but not related to the meniscus opening a new window of opportunity to study the role of the meniscus in the disease. Thus, some of them are potential candidates for further targeted studies since they are involved in pathways associated with the natural course of OA (Table 3) [[11], [12], [13], [14], [15], [16], [17], [18], [19], [20], [21], [22], [23], [24], [25], [26], [27]]. Specifically, we found CILP and extracellular superoxide dismutase [Cu–Zn] (SOD3) decreased in end-stage OA. The alteration in protein levels observed for CILP and SOD3 in the meniscal tissue is also confirmed in the synovial fluid from the same study individuals [21]. This fact supports the evidence that CILP and SOD3 are released into synovial fluid from the damaged meniscal tissue in OA.

**Table 3:** Potential role in OA of the proteins altered in the meniscus from patients with end-stage OA group.

Gene	Protein ID	Protein name	Potential role in OA
APOD	P05090	Apolipoprotein D	Lower levels of human APOD in cartilage, synovium, and serum were previously correlated with the severity of knee OA [11,13]. We reported lower levels of APOD in end-stage OA for the first time in the meniscus.
DPP4	P27487	Dipeptidyl peptidase 4	Recently, DPP4 was described as surface marker of senescent chondrocytes and a predictor of OA progression in synovial fluid [14]. We found lower abundance of DPP4 in end-stage OA samples, and potentially in the group with mild signs of joint damage. We hypothesized that DPP4 is gradually released from the damaged tissue to the synovial fluid.
B2M	P61769	Beta-2-microglobulin	B2M serum levels of OA patients were higher than the controls [15], whereas we found the opposite trend in the meniscal tissue.
NRP-1	O14786	Neuropilin-1	The overexpression of NRP-1 reduces inflammation, apoptosis and ECM degradation [16]. We found NRP-1 decreased in OA as compared to healthy controls which may worsen joint damage
CILP	O75339	Cartilage intermediate layer protein 1	Increased levels of CILP were found in the secretome of OA articular cartilage [17], which correlated with our findings. We reported a gradual decrease of CILP in the meniscus with the joint damage.
PRELP	P51888	Prolargin	Increased levels of PRELP were found in the secretome of OA articular cartilage [17]. We reported a gradual decreased of PRELP in the meniscal tissue with the joint damage. This could indicate that the protein is gradually being lost from the meniscus.
SOD3	P08294	Extracellular superoxide dismutase [Cu–Zn]	SOD3 which plays a crucial role in the maintenance of the cartilage homeostasis by the control of ROS was found decreased after OA incidence and proposed as drug target candidate in OA [18]. We found lower levels of SOD3 in end-stage OA meniscus as compared to healthy controls.
MFAP5	Q13361	Microfibrillar-associated protein 5	MFAP5 expression was associated with cartilage degeneration and OA severity [19]. We found decreased levels of MFAP5 with the gradual joint damage progression.
IGFBP2	P18065	Insulin-like growth factor-binding protein 2	Decreased in the synovial fluids from dogs with secondary OA after a cruciate ligament rupture [20]. We found lower levels of IGFBP2 in the human meniscus of end-stage OA as compared to healthy control and the opposite trend was found in human synovial fluid, making IGFBP2 a novel biomarker candidate for OA severity.
DCD	P81605	Dermcidin	DCD was found increased in the synovial fluid from patients with early knee OA [21]. Interestingly, DCD levels decreased in end-stage OA accordingly to our results. Decreased levels in the menisci could suggest a release into synovial fluid.
GYG1	P46976	Glycogenin-1	GYG1was implicated in OA pathogenesis by the interaction with asparagine [22]. We did not find an alteration in the GYG1 protein levels in patients with mild signs of joint damage whereas a decrease was found in end-stage OA.
BCAT1	P54687	Branched-chain-amino-acid aminotransferase, cytosolic	BCAT1 promotes osteoclast maturation by regulating branched-chain amino acid metabolism. The Branched chain amino acids metabolic pathway is altered because of either joint injury or articular cartilage pathology [23]. The BCAT1 deficit found in end-stage OA may reflect the crosstalk between different knee tissues during the OA process.
IGFBP6	P24592	Insulin-like growth factor-binding protein 6	IGFBP6 is downregulated in mechanically stressed aged human cartilage explants as compared to control [24]. We found a 3-fold decrease of IGFBP6 in the medial meniscus of end-stage OA patients compared to healthy controls.
SEMA3B	Q13214	Semaphorin-3B	SMA3B decreases the migratory and invasive capacities of FLS in RA and suppresses expression of matrix metalloproteinases suggesting a protective role of SEMA3B in a mouse model of arthritis [25]. The lower levels of SEMA3B detected in end-stage OA could promote the overexpression of metalloproteinases and cell migration.
VIT	Q6UXI7	Vitrin	The transition from prehypertrophic to hypertrophic chondrocytes in the growth plate is marked by a 10-fold decrease in VIT mRNA. This tightly regulated expression signifies a potential role in maintaining ECM structure in the cartilage matrix, making vitrin a novel candidate for human chondrodysplasias and an important target for further analysis in vivo [26]. Our proteomic data in medial meniscal tissue revealed a 3-fold decrease in VIT protein levels in end-stage OA compared to healthy controls.
PTPRS	Q13332	Receptor-type tyrosine-protein phosphatase S	PTPRS acts as a receptor of PG in the ECM. PTPRS is highly expressed on fibroblast-like synoviocytes where its function is regulated by an interaction with the heparan sulphate transmembrane PG. Lower levels of PTPRS may be the cause of increased levels of GPC6 detected in the group with mild signs of joint damage in our study. An increment of catabolic effects has been observed when adding heparan sulphate from OA cartilages possibly due to the loss of cell adhesion properties [27].

ECM: extracellular matrix; FLS: fibroblast-like synoviocytes; OA: osteoarthritis; PG: proteoglycans; RA: rheumatoid arthritis; ROS: reactive oxygen species.

A previous proteomic comparison of OA and healthy human menisci using a DIA MS/MS approach suggest robust results for those proteins clearly modulated in the meniscus of end-stage OA patients (see also Supplementary table 6) [8]. Both studies showed peroxiredoxin-2 (PRDX2) increased and thrombospondin-1 (THBS1) decreased in end-stage OA for both meniscal compartments. The THBS1 levels were also found decreased in the synovial fluid of end-stage OA patients [21]. THBS1 was shown to induce regeneration of damaged cartilage. The fall of THBS1 levels observed in OA may reflects a homeostasis imbalance towards catabolic processes leading to joint destruction [28].

Although the majority of identified proteins did not substantially differ in abundance between mild and control groups, we detected relevant differences (fold change>2; p-value< 0.05) for some specific proteins. In the mild^med^ group the levels of CCT6A, EIF4B, and SPEE were higher and QSOX1 lower as compared to healthy controls. The differential expression levels of the increased proteins in the mild^med^ group remains constant in the end-stage OA group whit the exception of QSOX1 which is even lower in end-stage OA patients. The trend exhibited by these proteins may reveal a role in OA from the early disease stages.

CCT6A is a component of the chaperonin-containing T-complex (TRiC) essential for cell viability and responsible for the proper folding of cytosolic proteins from the chondrocyte cytoskeleton like actin or tubulin [19]. EIF4B is required for the proper binding of mRNA to ribosomes. The deficit of EIF4B was reported to enhance the expression of inflammatory factors and chemokines implicated in the recruitment and activation of neutrophils and macrophages. The upregulation of EIF4B in the mild group may be involved in the onset of a knee OA inflammatory phenotype [29]. However, we did not detect relevant changes at protein levels of inflammatory factors in the mild group to support this hypothesis. Spermidine synthase (SPEE) is responsible for the conversion of putrescine to spermidine a polyamine with antioxidant and anti-inflammatory effects which promotes autophagy. Eisenberg and collaborators indicated that supplementation with exogenous spermidine reduces age-related oxidative protein damage in mice. Therefore, they suggest spermidine as an anti-ageing drug. In accordance with our results, Tootsi K and colleagues reported increased levels of spermidine in OA patients which might be caused by an extra activity of SPEE [30]. QSOX1 is secreted by fibroblasts and is responsible for the incorporation of laminin into the ECM to maintain cell-matrix adhesion properties [31]. Previous findings revealed decreased mRNA levels of laminin in patients with mild joint damage compared to healthy controls which could be caused by the decrease of QSOX1 from early disease stages herein reported [32]. However, we found laminin Subunit Gamma 1 (LAMC1) and Laminin subunit alpha-5 (LAMA5) increased in end-stage OA patients. The specifical function of QSOX1 is unknown but it has been identified as a potential novel ligand of the relaxin receptor (RXFP1) with a marked role in collagen metabolism by inducing the production of several matrix-metalloproteases [33,34]. Furthermore, higher serum levels of relaxin-2 (RLN2) have been recently reported in females with anterior cruciate ligament tears (ACL) compared to those without ACL injuries [35]. Thus, the role of QSOX1 is unclear.

In the lateral compartment of the mild group only the levels of GPC6 and C1QTNF8 increased. GPC6 is a cell-surface heparan sulphate proteoglycan (HSPG) involved in endochondral ossification and skeletal growth [27]. An increment of catabolic effects has been observed when adding heparan sulphate from OA cartilages which could explain by the loss of cell adhesion properties [36]. Lastly, C1QTNF8 belongs to a superfamily of proteins involved in the regulation of lipid metabolism and the immune-inflammatory response [37].

To highlight proteins that might be involved in the early stages of knee OA development we focused on those proteins which clearly follow a protein abundance pattern illustrated by a gradual change in protein levels from healthy to end-stage OA crossing mild joint damage group. We found a 42-protein panel following this trend. Among them, we found QSOX1 and GPC6, above described, for being the proteins most differentially expressed in the mild joint damage group. The ECM remodelling in OA is illustrated in our dataset by altered levels of several proteins comprised in the 42-panel including proteases, oxireductases and structural proteins (Supplementary Table 9) [38]. The clusterization of the interaction network showed altered levels of ECM related proteins undergoing destructive and tissue repair processes simultaneously. Most of the proteins assembled in a second cluster revealed an imbalance in the innate immune system possibly associated with the OA development [39]. Specifically, we found an overall pattern of a gradual decrease in levels of proteins related to the complement cascade from healthy to end-stage OA. A dysregulation of the complement system in OA could be promote by the recognition of damage-associated molecular patterns (DAMPs) produced during tissue damage. The products of extracellular matrix (ECM) breakdown, such as BGN, DCN, ACAN, and TNC (decreased in end-stage OA meniscus) or the gradual increment in the released of alarmins such as S100A8 from damaged cells could be act as DAMPs [40]. Previous studies found an increase in expression of complement-effector genes, and a decrease in expression of complement-inhibitor genes in a mouse model of OA induced by medial meniscectomy leading to the conclusion that complement system is overall activated in OA [41]. However, we found decreased levels of the complement effectors components C5, C7, C8, and C9 which may attenuate OA, whereas the lower levels of complement-inhibitors such as complement factor H (CFAH), C1-inhibiting factor (IC1) and clusterin (CLUS) identified in OA meniscus may worsened it. Then, we evidence the role of complement system in the pathogenesis of OA illustrated by an imbalance between protective and pathogenic responses.

Interestingly, a previous shotgun proteomic approach in synovial fluid [21] showed that changes in protein levels of CILP, keratin type II cytoskeletal 2 epidermal (KRT2), and transgelin (TAGLN) follow the same direction in synovial fluid and meniscal tissue. Conversely, the levels of insulin-like growth factor-binding protein 2 (IGFBP2) and lipopolysaccharide-binding protein (LBP) in synovial fluid followed the opposite pattern in the meniscal tissue. These findings may suggest that the meniscus is the source of the alterations detected in synovial fluid. Therefore, we proposed CILP, KRT2, TAGLN, IGFBP2 and LBP for further targeted proteomic studies regarding meniscal pathology and their role in OA even at early disease stages. Moreover, we reported lower levels of CRTAC1 in the meniscus of end-stage patients. Currently, CRTAC1 is one the most promising circulating biomarker in OA [42].

DIA-based MS allows for improved sensitivity and accuracy in protein quantification than DDA traditional approaches which translates to fewer missing values [43]. Although proteins with many missing values are not suitable for statistical analysis the profile expression could expose clear qualitative changes that are informative of the potential role of the protein in the disease.

In our study, S100A8 was not detected in any samples from the control group whereas the number of subjects where S100A8 was identified increased with the grade of meniscus degeneration until achieving a 100 ​% of identification in the end-stage OA group. The alarmin S100A8 is a mediator in the immune response outbreaks triggering inflammation. Alarmins are an emerging target of inflammation with therapeutic potential in OA. The preferred form for human S100A8 is the S100A8/S100A9 heterodimer. Higher levels of S100A8/S100A9 have been found in the serum, synovial fluid, and synovium of patients with OA and is positively associated with increased knee symptoms and cartilage degeneration even at early disease stages [44,45].

In contrast, CYTL1 was identified in most samples from control and mild groups whereas in the end-stage OA group the number of samples where CYTL1 was detected dramatically decreased. CYTL1 is required for cartilage homeostasis, and in accordance with our findings, CYTL1 (−/−) KO mice showed an increased cartilage destruction in a surgical OA model (destabilized medial meniscus) [46]. The gene was also found to be clearly downregulated in a porcine post-traumatic OA model indicating that this protein could be important for cartilage homeostasis [47].

### 4.1 Study limitations

Finally, we would like to point out some important limitations. Firstly, the proteins identified with the highest fold changes were carbonic anhydrase 1 (CA1), two subunits of haemoglobin (HBA and HBB), ankyrin-1 (ANK1) and catalase (CAT) described in the literature as blood contaminants [48]. Despite the complete meniscus was rinsed with buffer to clean the tissue surface before taking the plugs, it is unfeasible to discriminate whether the source of the plasma proteins is derived from the vascularization of the outer zone of the meniscus or the intra-articular bleeding after TKA surgery [8].

Secondly, the protein extraction method employed in this study is suboptimal for collagen extraction making the interpretation of their overall abundances difficult. Hydroxyproline, the most common collagen modification, was not included in the search, which limits the number of peptides identified, particularly for the fibrillar collagens.

The study design challenges the interpretation of the results obtained in the pairwise comparison. It is important to highlight the heterogeneity of the study participants in the mild group (all patients had medial mild signs of joint damage while 6 out of 13 also had lateral signs) making it difficult to extract conclusive information with such a limited sample size. We did not correct for multiple testing due to the exploratory nature of the study. We used mixed models for more efficient estimation and we report estimates from all comparisons made for transparency and to inform future potential meta-analyses [49].

Lastly, this study focused on OA included old and heavy individuals with slightly male dominant groups, so our findings are not generalized to other population.

## 5 Conclusion

The main original result from this work relies on the inclusion for the first time of individuals with mild signs of joint damage for the study of the meniscal role within the pathological process of knee OA. We reported minor differences between the proteome profile of the group with mild signs of joint damage and the healthy group while major differences were with the end-stage OA group. Further studies should be carried out to elucidate the relationship between the protein profiles found in meniscus with the molecular degeneration associated with knee OA. Despite the study limitations, the results provided would contribute with new insight into the processes involved in meniscus degeneration as a risk factor for the development of knee OA. Furthermore, we provide a novel panel of proteins, based on a pattern of progressive joint damage, to prioritize potential candidates for futures studies in the field.

## Author contributions

Conception and design: all co-authors; Provision of study materials and tissue preparation: ME, RPG, AT, NA and PÖ; Mass spectrometry analysis: RPG and PÖ; Statistical analysis: RPG and AT; Interpretation of results: all co-authors; Drafting of the article: RPG; Critical revision of the article: all co-authors; Final approval of the article: all co-authors.

## Funding

This work was also supported by grants from Fondo Investigación Sanitaria-Spain (PI20/00793 and PI22/01155) and RICORS-REI RD21/0002/0009), integrated in the National Plan for Scientific Program, Development and Technological Innovation 201–2020 and funded by the ISCIII-General Subdirection of Assessment and Promotion of Research - European Regional Development Fund (FEDER) “A way of making Europe”. This study was also supported by grants IN607A2021/07 and IN607D2020/10 from Xunta de Galicia. RPG was supported by inMOTION grant program from UDC-INDITEX. This work was supported by the European Research Council (ERC) under the European Union's Horizon 2020 research and innovation programme (grant agreement #771121), The Swedish Research Council, the ALF agreement between the Swedish government and the county, the Swedish Rheumatism Association, IngaBritt and Arne Lundberg's Research Foundation, the Alfred ​Österlund Foundation, the Olle Engkvist Foundation, the Greta & Johan Kock Foundation, Gustav V 80-year Birthday Foundation, The Foundation for Movement Disabilities in Skåne, the Anna-Greta Crafoord and the Crafoord Foundations.

## Conflict of interest

The authors do not have any conflict of interest.
