# Supplementary material for: Proteomic profiling of human menisci from mild joint degeneration and end-stage osteoarthritis versus healthy controls
Source: Osteoarthr Cartil Open. 2023 Nov 18;5(4):100417. doi: 10.1016/j.ocarto.2023.100417 (PMC10720269; doi:10.1016/j.ocarto.2023.100417)
Supplement: Multimedia component 1 [file mmc1.docx]

**Supplementary materials and methods**

**Materials**

N-Ethylmaleimide, 6-aminocaproic acid, benzamidine hydrochloride hydrate, dithiothreitol (DTT), iodoacetamide (IAA), ammonium bicarbonate (AMBIC), formic acid (FA), ethanol, UPLC grade acetonitrile and A (0.1% formic acid in water) and B (0.1% formic acid in acetonitrile) solutions for LC-MS were purchased from Sigma-Aldrich (St. Louis, USA). Guanidine hydrochloride (GdnHCl) and anhydrous sodium acetate (NaAc) were purchased from Merck (Darmstadt, Germany). Trypsin gold MS grade was purchased from Promega (Madison, WI). The water used in this study was purified using a MilliQ apparatus (Millipore, Billerica, MA). The Pierce Quantitative Colorimetric Peptide Assay, Pierce™ 660nm Protein Assay Kit and Pierce™ HeLa Protein Digest Standard were purchased from Thermo Fisher Scientific (Rockford). AcroPrep^TM^ 30K Omega Ultrafiltration plates were purchased from Pall Life Sciences (Ann Arbor, USA) and reversed-phase C18 tips AssayMAP^TM^ were purchased from Agilent (Cedar Creek, USA).

**Methods**

**Traceability conditions of the meniscal tissue**

The menisci from donors were obtained within 48 hours post-mortem and frozen at -80°C within 2 hours of extraction. For the end-stage knee OA group the menisci were retrieved during total knee replacement (TKR) surgery at Trelleborg Hospital in patients with primarily medial compartment tibiofemoral knee OA. The tissue was frozen at -20°C within 2 hrs after surgery and later transported to Lund on dry ice for final storage at -80°C. The knee joint cartilage was classified by the surgeon according to the Outerbridge classification system.

**Preparation of meniscal tissue for MS analysis**

For protein extraction, the tissue plugs were powdered in liquid nitrogen using a pestle and mortar technique. Tissue was weighed before and after pulverization to guarantee a total recovery. Proteins were extracted from the pulverized tissue using 15 volumes (15μL buffer/mg powder) of extraction medium (4M GdnHCl, 50mM NaAc, 100mM 6-aminocaproic acid, 5mM benzamidine, 5mM N-ethylmaleimide, pH 5.8), incubated on an orbital shaker at +4°C for 24h after which, extracted material was centrifuged at 13200 rpm at + 4 °C for 30 min. Protein concentration was determined in the supernatant using both the nanodrop instrument at 280 nm and the Protein Assay Kit at 660nm. Next, 50 μL of each sample was reduced using 10mM DTT, with shaking at +56°C for 30 min followed by alkylation with 40mM IAA for 1h at room temperature in the dark. The same amount of DTT was added again to neutralize the IAA excess. Extracts were precipitated overnight at +4°C to remove residual salts, using nine volumes of 96% ice-cold ethanol with 50mM NaAc and centrifugated at 13200 rpm at + 4 °C for 30 min. The supernatant was decanted, and the pellet was washed with 96% ethanol at -20°C for 4h. After centrifugation, the supernatant was removed, and the precipitate was dried in a SpeedVac.

Samples were dissolved in 100μL of 0.1M AMBIC and proteins were digested using 2 μg of Trypsin Gold with shaking at 37°C for 16h. Then, the digestion was stopped with a final concentration of 0.5M sodium chloride and 0.5 % formic acid (FA). The final peptide concentration was determined using Pierce Quantitative Colorimetric Peptide Assay according to the manufacturer’s instructions.

To remove longer peptides with glycosaminoglycan (GAG) chains, digested samples were filtered using a 30kD ultrafiltration plate and subsequently desalted in a Bravo robot (Agilent) using reversed-phase C18 columns. The elution was carried out in 50% acetonitrile (ACN) with 0.1% formic acid (FA). Samples were dried in the SpeedVac before MS analysis.

**LC-MS/MS method**

A heated ion transfer setting of 260°C was used for desolvation together with a spray voltage of +1800V. The online reversed-phase separation was performed using a flow rate of 300nL/min. For the DIA analysis, a binary gradient of 125min was used, starting with a 5min increase from 3% B to 7% B, then going to 20% B over 85min, further increasing to 30% B over 20min, and with a final 5min increase up to 90% B and kept at 90% B isocratic for 10min. For the MS settings, the MS1 scan (390-1210 m/z) was set to have a resolution of 120000 and automatic gain control (AGC) set at 3 x 10^6^ with 100 ms maximum ion injection time. This was followed by data-independent acquisition collision-induced dissociation MS2 scans at a resolution of 45000. A loop count of 26 with variable windows and 0.5Da overlap was used in the range of 350-1650 m/z [1].

**Data reproducibility**

### We used Bland-Altman approach to estimate repeatability coefficients for peptide quant from samples that were run twice [2].

The technical variation on the peptide quant data between the six duplicated samples (one sample per study group) showed a median repeatability coefficient of 0.89 (on log2 scale).

**References**

[1] E. Folkesson et al., "Proteomic characterization of the normal human medial meniscus body using data-independent acquisition mass spectrometry," J Orthop Res, vol. 38, no. 8, pp. 1735-1745, Aug 2020, doi: 10.1002/jor.24602.

[2] J. M. Bland and D. G. Altman, "Statistical methods for assessing agreement between two methods of clinical measurement," (in eng), *Lancet,* vol. 1, no. 8476, pp. 307-10, Feb 08 1986.
