# Supplementary material for: Proteomic profiling of human menisci from mild joint degeneration and end-stage osteoarthritis versus healthy controls
Source: Osteoarthr Cartil Open. 2023 Nov 18;5(4):100417. doi: 10.1016/j.ocarto.2023.100417 (PMC10720269; doi:10.1016/j.ocarto.2023.100417)
Supplement: Multimedia component 2 [file mmc2.docx]

**Supplementary Table 1. Study participants classification**. The Outerbridge score for cartilage and Pauli score for the meniscus of the study participants in the medial and lateral compartments.

**Supplementary Table 2. Results from the statistical analysis.** Differences (fold change log_2_) in terms of protein levels between the end-stage OA and mild group against the control, separately for medial and lateral compartments. Provided as an Excel table, n=1096.

**Supplementary Table 3.** **Proteins with no difference in abundance between any of the groups.** Proteins with no difference are defined by a confidence interval (CI) enclosed between -1 and 1 for all comparisons. Provided as an Excel table, n=213.

**Supplementary Table 4. Proteins with inconclusive results.** Proteins with inconclusive results are defined by a confidence interval (CI) width larger than 2.5 for at least one comparison. Provided as an Excel table, n=106.

**Supplementary Table 5.** **Proteins potentially regulated in either end-stage OA or mild OA groups in comparison with the control**. Provided as an Excel table, n=777.

**Supplementary Table 6. Proteins altered in the end-stage OA group as compared to healthy controls.** Proteins which altered levels (FC; fold change log_2_ ≥ 1; p-value< 0,05) comparing end-stage OA and healthy human menisci matched between two independent studies.

**Supplementary Table 7. Pathway enrichment analysis performed in STRING.** Pathways most strongly represented in the two independent cluster identified in the set of proteins following the protein abundance pattern (n=247).

**Supplementary Table 8**. **Potential qualitative changes in proteins with a pattern of missing data**. Provided as an Excel table, n=38.

**Supplementary Table 9.** Proteins following the protein abundance pattern restricted to those with differences (value of 1.5 in the CI) in the mild^med^ group classified by their biological function.
